# 

**Published:** 1877-11

**Authors:** 


### Books and Pamphlets Received.

Ziemssen's Cyclopœdia. Vol. XVI. Diseases of the Locomotive Apparatus and General Anomalies of Nutrition. New York: Wm. Wood & Co., 1877. Buffalo: J. H. Matteson.

Forensic Medicine and Toxicology. By W. Barthurst Woodman, M. D. F.R.C.P., and C. M. Tidy, M. B., F.C.S. Illustrated. Philadelphia: Lindsay & Blakiston, 1877. Buffalo: T. H. Butler.

Lectures on Practical Surgery. By H. H. Toland, M. D. Philadelphia: Lindsay & Blakiston, 1877. Buffalo: T. H. Butler.

An Index of Diseases and their Treatment. By T. H. Tanner, M. D. F.L.S. Philadelphia: Lindsay & Blakiston, 1877. Buffalo: T. H. Butler.

The Practitioner's Reference Book. By Richard J. Dunglison, M. D. Philadelphia: Lindsay & Blakiston, 1877. Buffalo: T. H. Butler.

A Treatise on the Ear. By Charles H. Burnett, A. M., M. D. Philadelphia: Henry C. Lea, 1877. Buffalo: T. H. Butler.

Biology. By Joseph Cook. Boston: James R. Osgood & Co., 1877.

Outlines of Modern Organic Chemistry. By C. Gilbert Wheeler. New York and Chicago: A. S. Barnes & Co., 1877.

Ziemssen's Cyclopœdia. Vol. V. *Reviewed in Vol. XV. of this Journal.*

Wood's Physician's Vade-Mecum and Visiting List. By H. C. Wood, M. D. Philadelphia: J. B. Lippincott & Co.

Transactions of the Medical Association of the State of Alabama. Thirteenth Session, 1877.

The London Lancet, August 1877.

Defects of Hearing the result of Hypertrophied Tonsils. A. W. Calhoun, M.D.

Popular Science Monthly, November, 1877. The Supplement Nos. V. and VI., 1877.

Catalogue of Medical Publications. Henry C. Lea.

Transactions of the International Medical Congress at Philadelphia, 1876. Philadelphia, printed for the Congress, 1877,

### Contents of November Number.

### ORIGINAL.

THE BUFFALO

### Medical and Surgical Journal

PUBLISHED MONTHLY,

Containing Original Articles, Reports of Medical Societies and Hospitals, Editorial, Reviews, Correspondence, Army News, etc., etc; including the usual variety of Medical Periodical Publications. Specimen copies sent on application

Address Buffalo Medical & Surgical Journal, Buffalo, N. Y.

TERMS OF SUBSCRIPTION, $3.00 PER YEAR, IN ADVANCE.

All communications for publication shoud be sent before the twentieth (20th) of each month, or they will of necessity lie over until the next number. No change can be made in advertisements after the twenty-fifth. Address, Buffalo Medical and Surgical Journal, 978 Main Street.

Correspondence and original articles are solicited. Publication is no evidence that the views of the author are endorsed.

